# ECU convention 2017 research presentations: Limassol, Cyprus. 25-27 May 2017

**DOI:** 10.1186/s12998-017-0166-7

**Published:** 2017-12-06

**Authors:** Alice Kongsted, Lise Hestbaek, Carlo Ammendolia, Pierre Côté, Danielle Southerst, Michael Schneider, Brian Budgell, Claire Bombardier, Gillian Hawker, Y. Raja Rampersaud, Corinne Minder, Cynthia Peterson, B. Kim Humphreys, Halldór Fannar Gíslason, Jari Kullervo Salminen, Linn Sandhaugen, Andreas Stenseth Storbråten, Renske Versloot, Inger Rouge, Dave Newell, Ellen Aartun, Hainan Yu, Pierre Côté, Erik Poulsen, Glaucia H. Goncalves, Ewa M. Roos, Jonas B. Thorlund, Carsten Juhl, Andreas Eklund, Irene Jensen, Malin Lohela-Karlsson, Jan Hagberg, Lennart Bodin, Charlotte Lebouf-Yde, Alice Kongsted, Iben Axén, Kristina Boe Dissing, Jan Hartvigsen, Christopher Williams, Steven Kamper, Eleanor Boyle, Niels Wedderkopp, Lise Hestbæk, Michael L. Meier, Petra Schweinhardt, Kim Humphreys, Amy Miller, Joyce Miller, Amy Miller, Joyce Miller, Amy Miller, Joyce Miller, Alison Taylor, Sue Way, Brigitte Wirth, Petra Schweinhardt, Kim Humphreys, Bruno A. P. Alvarenga, Marcelo B. Botelho, Jerusa P. R. Lara, António P. Veloso, Cecilia Bergström, Margareta Persson, Ingrid Mogren, B. Janine Thöni, Cynthia Peterson, B. Kim Humphreys, David Guillén, Cynthia Peterson, B. Kim Humphreys, Daniel Heritage, Joyce Miller, David Byfield, Annie Newsam, David Byfield, Mehmet Toprak, Hasan Kerem Alptekim, Doruk Turhan, Hazel Mellars, Joyce Miller, Jacqueline Rix, Philip Dewhurst, Jacqueline Rix, Caroline Cooke, David Newell, Joel Alcantara, Jeanne Ohm, Junjoe Alcantara, Joel Alcantara, Jeanne Ohm, Junjoe Alcantara, Jonathan Field, Dave Newell, Heather Hanson, Joyce Miller, Hiew Mandy, Lo Derek, Mok Zicheng, Tee Yun Han, Miller Joyce, Signe Fuglkjær, Kristina Boe Dissing, Lise Hestbæk, P. Schweinhardt, B. Wirth, G. Peterson, B. K. Humphreys, Tim L. Raven, Lise R. Lothe, Torsten Eken, Rick Serola

**Affiliations:** 1 Nordic Institute of Chiropractic and Clinical Biomechanics, University of Southern Denmark, 5230 Odense M, Denmark; 2 Department of Sports Science and Clinical Biomechanics, University of Southern Denmark, 5230 Odense M, Denmark; 3 Institute of Health Policy, Management and Evaluation, University of Toronto, Toronto, Canada; 4 Rebecca MacDonald Centre for Arthritis & Autoimmune Disease, Mount Sinai Hospital, Toronto, Canada; 5 Dalla Lana School of Public Health, University of Toronto, Toronto, Canada; 6 University of Ontario Institute of Technology, Oshawa, Ontario Canada; 7 Canadian Memorial Chiropractic College, Toronto, Canada; 8 Department of Physical Therapy, University of Pittsburgh, Pittsburgh, PA USA; 9 Department of Medicine, Division of Rheumatology, University of Toronto, Toronto, Canada; 10 Department of Medicine, University of Toronto, Toronto, Canada; 11 Department of Orthopedics, Toronto Western Hospital, University Health Network, Toronto, Canada; 12 Chiropractic Medicine Department, Faculty of Medicine, Orthopaedic University Hospital Balgrist, University of Zurich, Zürich, Switzerland; 13 Anglo European College of Chiropractic, 13-15 Parkwood Road, Bournemouth, BH5 2DF England; 14 UOIT-CMCC Centre for Disability Prevention and Rehabilitation, University of Ontario Institute of Technology, 2000 Simcoe Street North, Oshawa, ON L1H 7K4 Canada; 15 Department of Sports Science and Clinical Biomechanics, University of Southern Denmark, Odense M, Denmark; 16 Department of Physical Therapy, Federal University of Sao Carlos, Sao Carlos, Brazil; 17 Department of Rehabilitation, Copenhagen University Hospital, Herlev and Gentofte, Denmark; 18 The Unit of Intervention and Implementation Research for Worker Health - The Institute of Environmental Medicine, Karolinska Institutet, Stockholm, Sweden; 19 The Nordic Institute of Chiropractic and Clinical Biomechanics, Campusvej, 55 5230 Odense M, Denmark; 20 The Research Department, Spinecenter of Southern Denmark, Hospital Lillebælt, Østre Hougvej 55, DK-5500 Middelfart, Denmark; 21 Department of Sports Science and Clinical Biomechanics, Faculty of Health Sciences, University of Southern Denmark, Campusvej 55, DK-5230 Odense M, Denmark; 22 Nordic Institute of Chiropractic and Clinical Biomechanics, Campusvej 55, DK-5230 Odense M, Denmark; 23 Hunter Medical Research Institute, School of Medicine and Public Health, University of Newcastle, Callaghan, NSW Australia; 24 Hunter New England Population Health, Hunter New England Local Health District, Longworth Ave, Wallsend, NSW Australia; 25 The George Institute for Global Health, Level 3, 50 Bridge St, Sydney, NSW 2000 Australia; 26 Dalla Lana School of Public Health, University of Toronto, 155 College St, Toronto, ON M5T 3M7 Canada; 27 Institute of Regional Health Services Research, University of Southern Denmark, Winsloewparken 193, DK-5000 Odense C, Denmark; 28 Sports Medicine Clinic, Orthopaedic Department Hospital of Lillebaelt, Østre Hougvej 55, DK-5500 Middelfart, Denmark; 29 Interdisciplinary Spinal Research, Department of Chiropractic Medicine, University Hospital Balgrist, Zurich, Switzerland; 30 Alan Edwards Center for Research on Pain, McGill University, Montreal, Canada; 31 Bournemouth University, Poole, UK; 32 Anglo-European College of Chiropractic (AECC), Bournemouth, UK; 33 Bournemouth University, Poole, UK; 34 Anglo-European College of Chiropractic (AECC), Bournemouth, UK; 35 Bournemouth University, Poole, UK; 36 Anglo-European College of Chiropractic (AECC), Bournemouth, UK; 37 Interdisciplinary Spinal Research, Department of Chiropractic Medicine, University Hospital Balgrist, Zurich, Switzerland; 38 Lisbon University, FMH, Human Kinetic Faculty, 1499-002 Lisbon, Portugal; 39 University of Bahia, Medicine Faculty, Salvador, 40026-010 Brazil; 40 Federal University of Paraná, Curitiba, 80210-132 Brazil; 41 Lisbon University, FMH, Human Kinetics Faculty, Biomechanics and Functional Morphology Laboratory, 1499-002 Lisbon, Portugal; 42 Department of Clinical Sciences, Obstetrics and Gynecology, Umeå University, Umeå, Sweden; 43 Department of Nursing, Umeå University, Umeå, Sweden; 44 Department of Chiropractic Medicine, Faculty of Medicine, Orthopaedic University Hospital Balgrist, University of Zürich, Zürich, Switzerland; 45 Department of Chiropractic Medicine, Faculty of Medicine, Orthopaedic University Hospital Balgrist, University of Zürich, Zürich, Switzerland; 46 Anglo-European College of Chiropractic (AECC), Bournemouth, UK; 47 Welsh Institute of Chiropractic, University of South Wales, Pontypridd, UK; 48 Welsh Institute of Chiropractic, University of South Wales, Pontypridd, UK; 49 Mehmet Toprak, Research Assistant, Bahcesehir University, Istanbul, Turkey; 50 Hasan Kerem Alptekin, Assistant Professor, Bahcesehir University, Istanbul, Turkey; 51 Doruk Turhan, Istanbul Kemerburgaz University, Istanbul, Turkey; 52 Anglo-European College of Chiropractic (AECC), Bournemouth, UK; 53 Anglo-European College of Chiropractic (AECC), Bournemouth, UK; 54 Anglo-European College of Chiropractic (AECC), Bournemouth, UK; 55 The International Chiropractic Pediatric Association and Research Consultant, Sherman College of Chiropractic, Spartanburg, South Carolina USA; 56 The International Chiropractic Pediatric Association, Sherman College of Chiropractic, Spartanburg, South Carolina USA; 57 Private Practice of Chiropractic, Manila, Philippines; 58 The International Chiropractic Pediatric Association and Research Consultant, Sherman College of Chiropractic, Spartanburg, South Carolina USA; 59 The International Chiropractic Pediatric Association, Sherman College of Chiropractic, Spartanburg, South Carolina USA; 60 Private Practice of Chiropractic, Manila, Philippines; 61 Back2Health, Petersfield, UK; 62 Anglo-European College of Chiropractic (AECC), Bournemouth, UK; 63 Anglo-European College of Chiropractic (AECC), Bournemouth, UK; 64 Anglo-European College of Chiropractic (AECC), Bournemouth, UK; 65 Department of Sports Science and Clinical Biomechanics, Faculty of Health Sciences, University of Southern Denmark, Campusvej 55, DK-5230 Odense M, Denmark; 66 Nordic Institute of Chiropractic and Clinical Biomechanics, Campusvej 55, DK-5230 Odense M, Denmark; 67 University Hospital Balgrist, Zürich, Switzerland; 68 Zurich University, Zürich, Switzerland; 69 Department of Anaesthesiology, Oslo University Hospital (OUH), University of Oslo, Oslo, Norway; 70 Founder and Co-CEO of Serola Biomechanics, Inc, Loves Park, Illinois USA

## Oral research paper presentations

### O-01 Care seeking patterns during one year after visiting a chiropractor

#### Alice Kongsted^1,2^, Lise Hestbaek^1,2^

##### ^1^Nordic Institute of Chiropractic and Clinical Biomechanics, University of Southern Denmark, 5230 Odense M, Denmark; ^2^Department of Sports Science and Clinical Biomechanics, University of Southern Denmark, 5230 Odense M, Denmark

###### **Correspondence:** Alice Kongsted (a.kongsted@nikkb.dk)

**Background**

Low back pain (LBP) is a very frequent cause for care seeking and often a long-lasting condition. However, little is known about individuals’ care seeking patterns over time. Therefore the objectives of this study were 1) to describe care seeking patterns 1 year after an initial chiropractic consultation, and 2) to examine how care seeking patterns related to pain intensity trajectories.

**Methods**

Danish chiropractic clinics recruited 947 adult patients at the initial consultation for LBP. From this cohort, 617 (65%) responded to questions about care seeking within the last 2 weeks at all of the follow-ups 2 weeks, 3 months and 12 months after inclusion. Based on these responses, we described care seeking patterns and investigated if care seeking was associated with trajectories of LBP severity. The LBP trajectories had previously been derived from weekly measures of pain intensity collected via SMS-tracking.

**Results**

Care seeking after the initial visit was reported by 95% of the patients. The most frequent care seeking pattern (51%) was to report care seeking at the 2-weeks follow-up and not later, 29% reported care seeking only 2 weeks and 3 months after the initial consultation, and 11% reported care seeking at all follow-up time points. Of those seeking care after 2 weeks, 3 months and 1 year, 98%, 76% and 50% respectively had chosen to see a chiropractor at those time points. At 1-year follow-up 18% of care seekers had visited a general practitioner and 27% a physiotherapist. Care seeking was associated with LBP trajectories: Most people who recovered from pain stopped care seeking, and those with persistent severe pain did most frequently seek care at all follow-ups. However, those with mild to moderate LBP had more diverse care seeking behaviours. For example in LBP trajectories of on-going moderate pain, 25% did not report care seeking after 2-weeks, and another 25% reported care seeking at all time points.

**Conclusions**

Most patients consulting Danish chiropractors for LBP are seen again within two weeks of the initial consultation and do not report continued care seeking after 3 months. One year after the initial visit, around half of the care seekers see other health care providers for their LBP instead of a chiropractor. Care seeking is related to LBP symptoms, but not in a uniform way, and it should be investigated why some people with on-going LBP continue to seek care while others do not.

The study was presented to the local ethics committee. The committee found that it did not need approval, since there was no intervention involved, which is in line with Danish law (Danish National Committee on Biomedical Research Ethics. Guidelines about Notification. (http://www.nvk.dk/english/act-on-research).

The project was approved by the Danish Data Protection Agency (J-no. 2004-41-4763 and J-no. 2010-41-5163).

### O-02 Non-operative management of lumbar spinal stenosis: a randomized controlled trial comparing a comprehensive vs. a self-directive approach

#### Carlo Ammendolia^1,2^, Pierre Côté^1,3,4^, Danielle Southerst^5^, Michael Schneider^6^, Brian Budgell^5^, Claire Bombardier^7,8^, Gillian Hawker^7,8^, Y. Raja Rampersaud^9^

##### ^1^Institute of Health Policy, Management and Evaluation, University of Toronto, Toronto, Canada; ^2^Rebecca MacDonald Centre for Arthritis & Autoimmune Disease, Mount Sinai Hospital, Toronto, Canada; ^3^Dalla Lana School of Public Health, University of Toronto, Toronto, Canada; ^4^University of Ontario Institute of Technology, Oshawa, Ontario, Canada; ^5^Canadian Memorial Chiropractic College, Toronto, Canada; ^6^Department of Physical Therapy, University of Pittsburgh, Pittsburgh, PA, USA; ^7^Department of Medicine, Division of Rheumatology, University of Toronto, Toronto, Canada; ^8^Department of Medicine, University of Toronto, Toronto, Canada; ^9^Department of Orthopedics, Toronto Western Hospital, University Health Network, Toronto, Canada

###### **Correspondence:** Carlo Ammendolia (cammendolia@mtsinai.on.ca)

**Introduction**

Degenerative lumbar spinal stenosis causing neurogenic claudication is a leading cause of pain, disability and loss of independence in older adults. Effective non-surgical approaches are unknown. The study objective is to compare outcomes following comprehensive care that included manual therapy to self-directed care.

**Methods**

Consenting participants with degenerative lumbar spinal stenosis causing neurogenic claudication and limited walking ability (<30 minutes) were randomized to a 6-week comprehensive program that included twice weekly manual therapy or self-directed care following a single educational session. Both groups received a pedometer and exercise manual and video with instructions on daily home exercises and self-management strategies. Follow-up assessments were performed at 8 weeks, 3, 6 and 12 months. The primary outcome was the between group mean difference in walking distance from baseline to 12 months using the self-paced walking test assessed by a blinded assessor. Secondary outcomes included the mean difference in the functional and symptom scales of the Zurich Claudication Questionnaire (ZCQ), the Oswestry Disability Index (ODI), the ODI walking subscale, and the Numeric Pain Scale (NPS) for low back and leg pain. Intention-to-treat analysis was performed for all outcomes.

**Results**

Fifty-one participants were randomized to the comprehensive and 53 to the self-directed group. The adjusted mean difference in walking distance in the comprehensive group verses the self directed group at 8 weeks was 345.4 metres (m) (95% confidence interval (CI), 150.0 to 540.7), at 3 months, 304.1 m (95% CI, 77.9 to 530.3), at 6 months, 421.0 m (95%CI, 181.4 to 660.6) and at 12 months, 436.8m (95% CI, 200.3 to 673.4). At 12 months, 81% and 74% of participants in the comprehensive group achieved at least 30% and 50% improvement in walking distance respectively compared to 59% and 50% of participants respectively in the self-directed group. At 12 months all secondary outcomes showed significant improvement from baseline in both groups with the comprehensive group demonstrating significantly greater improvement in the ZCQ functional scale, -0.27 (95% CI, -0.49 to -0.04) and the composite ZCQ score, -48 (95% CI, -0.90 to -0.06).

**Conclusions**

Both approaches showed significant improvement in walking distance at each follow-up period with the comprehensive approach demonstrating superior improvement. The superior benefits of the comprehensive approach were sustained through 12 months even after the active intervention was terminated. It appears that manual therapy combined with one-on-one instruction on daily exercises and self-management strategies can provide better outcomes than a self-directed approach.

Mount Sinai Hospital Research Ethics Board No. 14-0020

ClinicalTrials.gov number, NCT02592642

### O-03 Factors associated with pain medication use in chiropractic low back pain patients and relation to treatment outcomes: a prospective outcomes study

#### Corinne Minder, Cynthia Peterson, B. Kim Humphreys

##### Chiropractic Medicine Department, Faculty of Medicine, Orthopaedic University Hospital Balgrist, University of Zurich, Zürich, Switzerland

###### **Correspondence:** Cynthia Peterson (xraydcpeterson@yahoo.ca)

**Study Objectives**

Pain medication prescription as a treatment option for chiropractors remains a controversial topic internationally. Some regions have obtained limited prescription rights for chiropractors. Switzerland is one of these countries. Therefore, the objectives of this study are: 1) to explore baseline patient factors associated with the use of pain medications; 2) to determine if there are differences in treatment outcomes between patients who do and do not use pain medications.

**Methods and Materials**

This is a prospective cohort outcome study including 1077 adult patients presenting with low back pain of any duration. Inclusion criteria required that patients had no chiropractic therapy in the 3 months prior to presentation. Multiple chiropractors contributed patients with no restrictions made to treatment methods. Patients completed a baseline questionnaire including questions on pain medication use and demographic data. Baseline pain and disability levels were measured using the numerical rating scale (NRS) and Oswestry questionnaire (ODI). Treatment outcomes were collected at 1 week, 1 and 3 months using these same outcome measures. The primary outcome used the Patient’s Global Impression of Change scale collected at these same time points. Demographic factors were compared between pain medication users and non-users with the Chi2 test. The Mann-Whitney-U test compared baseline pain and disability scores between the 2 groups and scores at all follow-up time points. The proportion of patients ‘improved’ at all time points was compared using the Chi2 test.

**Results**

Acute patients were more likely to take pain medication (p = 0.0001), as well as patients with radiculopathy (p = 0.0001), smokers (p = 0.008) and patients reporting below average general health (p = 0.0001). Medication users had higher baseline pain (p = 0.0001) and disability (p = 0.0001) scores. However, there were no significant differences in the proportion of patients ‘improved’ at any time point between the two groups. There was also no significant difference in NRS scores except at the 1 week time point (p = 0.0001). The ODI total scores remained higher in pain medication users at all time points but this was less than a 1 point difference at 3 months (p = 0.022).

**Conclusions**

Pain medication users are more likely to be acute, present with radiculopathy, smoke and have below average health status. There is no difference in the primary outcome of ‘improvement’ between users and non-users of pain medication at any time point.

KEK (Canton of Zurich ethics committee) Balgrist hospital ethics committee.

### O-04 The shape of chiropractic in Europe survey: a preliminary comparative analysis of two self selected identities

#### Halldór Fannar Gíslason, Jari Kullervo Salminen, Linn Sandhaugen, Andreas Stenseth Storbråten, Renske Versloot, Inger Rouge, Dave Newell

##### Anglo European College of Chiropractic, 13-15 Parkwood Road, Bournemouth BH5 2DF, England

###### **Correspondence:** Dave Newell (dnewell@aecc.ac.uk)

**Background**

The chiropractic profession has historically had diverse opinions concerning paradigms of health care approaches and the science or otherwise that underpins such beliefs. McGregor et al (2013) reported that adherence to orthodox and unorthodox paradigms as defined by 5 statements, were associated with types of practice behaviour considered outside of accepted evidence based guidelines in Canada. However, this type of investigation has not been repeated in other national jurisdictions and such relationships may be different.

**Methods**

A modified survey was disseminated amongst European chiropractors during early 2017. Dissemination was though an on-line platform with links to the survey being sent to all European chiropractic associations regardless of ECU membership and through the EAC. Social media was also used to disseminate the links to the survey.

**Results**

Analysis of 1132 respondent from chiropractors across Europe will be presented constituting approximately 16% of the profession. Stratification of beliefs into 5 categories representing a range of identities or paradigms from predominantly MSK focused (orthodox) focused to beliefs that subluxations are an impediment to health (unorthodox) was carried out based on respondents’ answers. Preliminary analysis restricted to orthodox and unorthodox categories revealed they constituted 47.7% (n=541) and 18.2% (n=206) of respondents respectively. When comparing the percentage of new patient’s chiropractors x rayed, 23% of the unorthodox group x rayed >50% of their new patients compared to 5% in the orthodox group. Those stating that x rays were used to identify subluxations constituted 33% of the unorthodox group compared to 2% in the orthodox group. Furthermore, respondents reporting >150 patient encounters per week in the unorthodox were double those in the orthodox group (22v11%). Lastly the proportion of those respondents disagreeing or strongly disagreeing with the statement “In general, vaccinations have had a positive effect on global public health” was 56.8% and 4.2 % respectively in unorthodox and orthodox categories.

**Conclusion**

Despite the limitations with generalisability in this survey, the proportion of respondents adhering to the different belief categories are remarkably similar to the randomly sampled survey in Canada (McGregor, 2013). In addition, and in parallel with Canadian chiropractors, this survey suggests that key practice characteristics in contravention of national radiation guidelines or opposition to evidence based public health policy are significantly more associated with non-orthodox views.

AECC ethics

The undergraduate projects are approved through an ethics process. Which is standard for higher education. No numbers are assigned. What the students have already sent you is their name tif stood of approval which is how we do ethics at the university. The ethics policy of the institution is on line.

### O-05 A scoping review of the literature on functioning and disability in manual medicine using the International Classification of Functioning, Disability and Health (ICF)

#### Ellen Aartun, Hainan Yu, Pierre Côté

##### UOIT-CMCC Centre for Disability Prevention and Rehabilitation, University of Ontario Institute of Technology, 2000 Simcoe Street North, Oshawa, ON L1H 7K4, Canada

###### **Correspondence:** Ellen Aartun (Ellen.Aartun@uoit.ca)

**Objectives**

Our first objective was to identify questionnaires used to measure functioning and disability in patients with low back pain who treated by manual medicine practitioners. Second, we aimed to link them to the International Classification of Functioning, Disability and Health (ICF) framework.

**Data sources and selection**

We systematically searched Medline, Embase, PsycINFO, and CINAHL from January 1, 2006 to December 31, 2015. Paired reviewers independently screened groups of 100 randomly selected citations from the retrieved citations. We included studies of patients with low back pain who received manual medicine (manipulation or mobilization). One reviewer extracted all background variables, baseline measurements and outcome measures from relevant studies. We divided the extracted data into constructs describing functioning and disability according to the ICF. Then, we linked the constructs to ICF categories using established linking rules. This process was repeated until we reached saturation, i.e., <5% difference in ICF categories.

**Results**

We retrieved 1995 citations and screened 200. We included 14 citations from the first round and 27 citations from the second round of screening. We extracted 21 unique questionnaires from the retrieved citations. The most commonly used questionnaires are Oswestry Disability Index, Roland-Morris Disability Questionnaire and 36-Item Short Form Health Survey (SF-36). In total, we identified 531 constructs describing functioning and disability in the questionnaires: body function (n=204), body structure (n=0), activities and participation (n=199), environmental factors (n=24) and personal factors (n=89). Some constructs were non-defined (n=14) or not covered by the ICF (n=1). Most items were linked to ICF chapters “b2” Sensory functions and pain (n=142), “d4” Mobility (n=73), “b1” Mental functions (n=55), “d5” Self-care (n=28) and “d6” Domestic life (n=28). In addition, beliefs about pain or life situation were relatively frequent (n=81) and linked to personal factors.

**Conclusion**

Questionnaires that are commonly used in manual medicine mainly focus on pain, beliefs about pain, psychological factors and activities such as walking, self-care and household tasks. Assessment of participation in social activities and environmental factors are not commonly investigated in the questionnaires. Future research needs to explore how these factors relate to the experiences of patients with low back pain who receive manual medicine.

The study was a scoping review of the literature and therefore, we did not need an ethical approval prior of the conduction of the study.

### O-06 Quantifying the risk of developing knee osteoarthritis following knee injury – a systematic review and meta-analysis

#### Erik Poulsen^1^, Glaucia H. Goncalves^2^, Ewa M. Roos^1^, Jonas B. Thorlund^1^, Carsten Juhl^1,3^

##### ^1^Department of Sports Science and Clinical Biomechanics, University of Southern Denmark, Odense M, Denmark; ^2^Department of Physical Therapy, Federal University of Sao Carlos, Sao Carlos, Brazil; ^3^Department of Rehabilitation, Copenhagen University Hospital, Herlev and Gentofte, Denmark

###### **Correspondence:** Erik Poulsen (epoulsen@health.sdu.dk)

**Study objectives**

To estimate the risk of knee osteoarthritis development following anterior cruciate ligament (ACL) injury, meniscal injury or a combined injury affecting both the ACL and meniscus.

**Methods and materials**

A systematic search was performed in MEDLINE, EMBASE, CINAHL, SPORTDiscus and Web of Science up to April 1st 2015. In addition, references from included publications and relevant systematic reviews were screened. Two authors independent screened title and abstract for eligibility followed by screening of full-text articles of eligible papers according to the following criteria: prospective or retrospective studies with ≥2-year follow-up after knee injury, ≥18 years old, un-injured contralateral knee or an un-injured group for comparison, outcome of knee OA defined by radiographs, symptoms or self-reported symptoms.

Risk of bias was assessed using the SIGN50 quality assessment tool for observational studies. Disagreement during eligibility, study inclusion and risk of bias assessment was resolved by consensus. Meta-analysis was applied based on the logarithmic transformed Odds Ratio (OR) of developing knee osteoarthritis in subjects with ACL injury, meniscal injury and combination of the two. Random effect models were applied as heterogeneity was expected in participants, follow-time and knee OA definitions. Inconsistency between study estimates was evaluated using the I-square statistics. Study registration: PROSPERO (ID: CRD42015016900).

**Results**

A total of 4559 papers were reviewed by title and abstract, 261 papers screened for full-text and 46 papers included in the meta-analysis. Knee OA diagnosis was based on knee radiographs in 96% of studies. For ACL injury, 12 studies were included (185.276 participants, mean age 28.0, 35% females) demonstrating an odds ratio (OR) with 95% confidence interval of 4.2 (2.4 – 7.6). For meniscal injury, 20 studies (6.211 participants, mean age 33.0, 23% females), OR of 5.8 (3.9 – 8.6) and for the combined ACL and meniscal injury type, 18 studies (1295 participants, mean age 25.5, 32% females), OR of 7.0 (4.8 – 10.4). The inconsistency between study estimates for the three different injury types were between 57-91%.

Risk of bias assessment rated two studies of high quality, 43 acceptable, and one study of unacceptable quality.

**Conclusion**

The risks of developing OA following either an ACL or meniscal injury are four and six times higher compared to developing OA in a non-injured knee. When sustaining a combined injury affecting both the ACL and meniscus, the risk is 7-fold. However, with large inconsistency of the estimates. Almost all studies included were of moderate quality.

### O-07 Prevention of low back pain: effect of chiropractic maintenance care as compared to symptomatic treatment – a pragmatic randomized clinical trial

#### Andreas Eklund^1^, Irene Jensen^1^, Malin Lohela-Karlsson^1^, Jan Hagberg^1^, Lennart Bodin^1^, Charlotte Lebouf-Yde^3^, Alice Kongsted^2^, Iben Axén^1,3^

##### ^1^The Unit of Intervention and Implementation Research for Worker Health - The Institute of Environmental Medicine, Karolinska Institutet, Stockholm, Sweden; ^2^The Nordic Institute of Chiropractic and Clinical Biomechanics, Campusvej 55 5230 Odense M, Denmark; ^3^The Research Department, Spinecenter of Southern Denmark, Hospital Lillebælt, Østre Hougvej 55, DK-5500 Middelfart, Denmark

###### **Correspondence:** Andreas Eklund (andreas.eklund@ki.se)

**Background**

Most individuals with non-specific low back pain (LBP) experience recurrent episodes, and for some, the pain becomes persistent. Prevention should therefore be targeted to prevent new episodes or to minimize the impact of the condition. Chiropractors have traditionally used a pre-planned manual treatment package, so called Maintenance Care (MC) with a preventive intent. The aim of this trial was to investigate the effect of MC as compared to symptomatic treatment on reducing recurrent and persistent LBP.

**Materials and Methods**

This was a multi-center randomized clinical trial conducted in Sweden. Individuals with recurrent or persistent LBP who responded well to manual treatment were assigned to either MC (treatment at regular intervals regardless symptoms) or to symptomatic treatment (where patients were treated only when consulting for pain). The trial was pragmatic in the sense that treatment content was decided by the clinicians. In the MC-arm, subjects were scheduled at 1-3 months intervals. In the control arm, subjects were told to consult when in pain. Subjects were followed with weekly text messages for 52 weeks to measure the primary outcome “number of days with bothersome LBP”, which was summarized for the 12 months.

**Results**

The MC group consisted of 163 subjects who reported 19.3 (95% CI: 4.9, 33.6) fewer days with bothersome LBP over the 12 month follow-up compared to the 154 subjects in the control group. The control group had an average of 7 visits during the study, compared to 5 visits in the control group.

**Conclusion**

MC, treatment on a regular basis regardless symptoms, is more effective than symptomatic treatment (after pain has recurred) in reducing the number of days with bothersome pain for patients with recurrent and persistent LBP.

**Acknowledgements**

This study was funded by the Institute for Chiropractic and Neuro-musculoskeletal Research, the European Chiropractors’ Union (project ID A13.02) and the Danish Chiropractic Research Foundation (grant number 11/148).

**Trial Registration**

Clinical trials.gov; NCT01539863, February 22, 2012

The ethical permission is 2007/1458-31/4 (from the regional ethics committee at Karolinska Institutet).

### O-08 Spinal pain in Danish school children – how often and how long? The Champs Study-DK

#### Kristina Boe Dissing^1^, Jan Hartvigsen^1,2^, Christopher Williams^3,4^, Steven Kamper^5^, Eleanor Boyle^1,6^, Niels Wedderkopp^7,8^, Lise Hestbæk^1,2^

##### ^1^Department of Sports Science and Clinical Biomechanics, Faculty of Health Sciences, University of Southern Denmark, Campusvej 55, DK-5230 Odense M, Denmark; ^2^Nordic Institute of Chiropractic and Clinical Biomechanics, Campusvej 55, DK-5230 Odense M, Denmark; ^3^Hunter Medical Research Institute, School of Medicine and Public Health, University of Newcastle, Callaghan, NSW Australia; ^4^Hunter New England Population Health, Hunter New England Local Health District, Longworth Ave, Wallsend, NSW Australia; ^5^The George Institute for Global Health, Level 3, 50 Bridge St, Sydney, NSW 2000, Australia; ^6^Dalla Lana School of Public Health, University of Toronto**,** 155 College St, Toronto, ON M5T 3M7, Canada; ^7^Institute of Regional Health Services Research, University of Southern Denmark, Winsloewparken 193, DK-5000 Odense C, Denmark; ^8^Sports Medicine Clinic, Orthopaedic Department Hospital of Lillebaelt, Østre Hougvej 55, DK-5500 Middelfart, Denmark

###### **Correspondence:** Kristina Boe Dissing (kbdissing@health.sdu.dk)

**Objectives**

The overall aim of this study was to describe the prevalence of spinal pain in 9-15 year-old Danish schoolchildren, over a three-year period. Specifically determining the characteristics of spinal pain in terms of frequency and duration.

**Methods**

A three-year prospective longitudinal cohort study following a cohort of 1400 school children. Parents received weekly text messages (SMS) inquiring about the child’s musculoskeletal pain.

**Results**

The results were presented separately for each study year (year 1, 2 and 3). The prevalence was 29%, 33% and 31% for year 1, 2 and 3 respectively, and increased with age, especially for lumbopelvic pain. Most children had few and short episodes with spinal pain, but 21%, 20% and 25% had three or more episodes during a study year and 16%, 17% and 17% of all episodes lasted for more than four weeks for year 1, 2 and 3 respectively.

**Conclusion**

This study confirms the relatively high prevalence of spinal pain in young people. Most episodes are brief, but there is a substantial group of children with frequent and long-lasting episodes of spinal pain indicating a need for action.

Ethics committee approval was obtained before the start of the project; ID S20080047, and registration in the Danish Data Protection Agency was made, as stipulated by Danish law J.nr. 2008-41-2240.

### O-09 Multivariate decoding of sensorimotor brain activity during lumbar pressure stimulation accurately identifies chronic low back pain

#### Michael L. Meier^1^, Petra Schweinhardt^1,2^, Kim Humphreys^1^

##### ^1^Interdisciplinary Spinal Research, Department of Chiropractic Medicine, University Hospital Balgrist, Zurich, Switzerland; ^2^Alan Edwards Center for Research on Pain, McGill University, Montreal, Canada

###### **Correspondence:** Michael L. Meier (michael.meier@balgrist.ch)

**Study objectives**

Aberrant cortical processing of nonpainful spinal sensory signals may be of particular importance in maintaining clinical pain in chronic low back pain (cLBP). Here we used functional magnetic resonance imaging (fMRI) in combination with machine learning methods to classify cLBP patients and healthy subjects by means of whole-brain activity patterns during nonpainful posterior-to anterior (PA) lumbar pressure stimulation. Due to the evidence of maladaptive cortical processes in cLBP patients we hypothesized to find the most predictive neural information differentiating the two groups in sensory/motor and limbic areas of the brain.

**Methods and material**

16 cLBP patients (11 females, mean age = 40,7, SD = 16.9) and 14 healthy controls (8 females, mean age = 37,5, SD = 10,35) participated in the study. Groups were gender- and age-matched. During fMRI acquisition, subjects were scanned in the prone position with their face lying on a modified stabilization pillow. A manual therapist applied a total of 54 controlled, nonpainful PA pressure stimuli of 4s duration at 3 different lumbar vertebrae (L1, L3, and L5) in a randomized order. Target pressure force was between 20N – 30N. Using a general linear model approach (implemented in SPM, v6685), individual beta images of the L1, L3 and L5 stimulations were generated and implemented in ProNTo (v2.0) for machine learning. The fMRI signal amplitude was normalized across all voxels so that brain activity mainly differed in its spatial distribution. Machine learning was performed by applying brain activity pattern recognition using a support vector machine which identifies a hyperplane that separates the two groups. Furthermore, a «leave one subject out» cross-validation was applied which ensured that the identified patterns were always tested on new, out-of-sample individuals.

**Results**

The applied pressure force was not significantly different between groups (two-sample t-test, t = 1.62, p > 0.05). Based on 10’000 permutations, the machine learning model demonstrated a between-group classification accuracy of 89.29% (p = 0.0001) with a specificity of 100% and sensitivity of 84.21%. The respective whole-brain voxel weight map indicated the most predictive voxels across the sensorimotor cortex including the bilateral postcentral gyrus and the supplemental motor area.

**Conclusion**

Using nonpainful PA pressure stimulation, the applied pattern recognition based method was able to predict group membership (cLBP vs healthy controls) with high accuracy. Strongest predictive voxels were found exclusively across the sensorimotor cortex which supports the evidence of sensory cortical reorganization processes in cLBP patients.

The study was approved by the Canton of Zurich’s Ethics Committee and was conducted in compliance with the declaration of Helsinki.

## Poster research paper presentations

### P-01 Incidence of tongue tie in 131 cases of suboptimal breastfeeding at the AECC and affiliated interdisciplinary breastfeeding clinic

#### Amy Miller^1^, Joyce Miller^2^

##### ^1^Bournemouth University, Poole, UK; ^2^Anglo-European College of Chiropractic (AECC), Bournemouth, UK

###### **Correspondence:** Amy Miller (amy.miller.chiro@gmail.com)

**Objectives**

Infant feeding difficulties are increasingly presented to the chiropractor, with growing evidence of musculoskeletal components playing a role in suboptimal breastfeeding. The incidence and presentation of tongue tie in this population is complex and not yet understood, with little clarity on the role of frenulotomy when a diagnosis of tongue tie is made. Hence, this study was carried out in order to gain baseline characteristics of tongue tie variables in this population with a view to directing further study.

**Methods and Material**

Data were collected as part of routine clinical information completed by mothers at initial presentation of their infant to the Anglo-European College of Chiropractic Teaching Clinic on the south coast of England.

**Results**

A total of 131 infants were included in this study. At the time of presentation to the AECC Clinic, 10% of infants had not previously been assessed for tongue tie, 51% had no tongue tie and 39% had been diagnosed with tongue tie. Of those diagnosed with a tongue tie, 12 (23%) had not been cut, 30 (59%) had been cut once, and 9 (18%) had been cut two or more times. Of the limited cases where information was provided regarding the perceived effect of the tongue tie cut, seven mothers felt there had been no improvement or worsening after frenulotomy and two mothers felt it had made a great improvement in feeding.

**Conclusions**

Although frenulotomy is routine when a diagnosis of tongue tie is made, it is questionable whether this is the appropriate management in every case. A total of 39% of infants in this study had a previous diagnosis of tongue tie. Of these, more than three quarters had undergone frenulotomy, and yet these infants were still presented to this clinic for the problem of sub-optimal breastfeeding. The design of this study cannot determine efficacy or effectiveness of care. Further, the numbers are too small to make any indications beyond possible trends. The only conclusions from this study are that first, tongue tie is a common problem in suboptimal breastfeeding, and second, that there is an unmet clinical need for clearer diagnostic criteria including differentiation of cases which would benefit from frenulotomy and those which would not. Alternative or additional treatment options should be explored for effectiveness, such as musculoskeletal care to release associated relevant tissues.

Ethical approval was granted from the AECC Research Ethics Subcommittee.

### P-02 Common musculoskeletal problems identified in 266 infants presented to an interdisciplinary breastfeeding clinic

#### Amy Miller^1^, Joyce Miller^2^

##### ^1^Bournemouth University, Poole, UK; ^2^Anglo-European College of Chiropractic (AECC), Bournemouth, UK

###### **Correspondence:** Amy Miller (amy.miller.chiro@gmail.com)

**Objectives**

Parents commonly seek multiple types of care for feeding problems for their babies. Evidence is emerging for the impact of musculoskeletal (MSK) dysfunction in infants with suboptimal breastfeeding, yet there is a deficit of research into common clinical practice in musculoskeletal health in infants. Research does implicate birth trauma as a key factor for the onset of MSK dysfunction in the new-born. The goal of this study was to assess Infants presented to an interdisciplinary chiropractic and midwifery breastfeeding clinic for any MSK dysfunctions, as well as to collect data on type of birth and feeding problems for each case. An aim of this study was to identify specific MSK dysfunctions which may be implicated in suboptimal breastfeeding.

**Methods and materials**

Standardised forms were utilised by chiropractic interns to gather data prospectively on infants presented by their mothers to the interdisciplinary breastfeeding clinic on the south coast of England between September 2015 and July 2016. These forms collected data on feeding abilities, birth type and any MSK problems in the infant. Descriptive data were analysed using Microsoft Excel.

**Results**

In total, 266 infants were part of the study. The mean age at presentation was 5.7 weeks, 52% of babies were male, 48% were female and the mean gestational age at birth was 39.7 weeks. Feeding difficulties were reported by all mothers and were most commonly difficulty attaching baby to the breast (54%), painful feeding (44%) and one-sided breastfeeding (35%). Birth was assisted in 64% of this population, with forceps deliveries more than twice as common compared to the local average. On examination, 65% of babies had dysfunction in the thoracic spine, 56% in the cervical spine and 44% in the sternocleidomastoid muscles. Other MSK problems were noted by the mothers in this population, including 43% unable to sleep supine, 45% with postural head preference and 29% demonstrated positional head deformation.

**Conclusion**

In this group of infants presented for suboptimal breastfeeding, MSK problems and assisted births were common. Considering that the cervical and thoracic spine along with the sternocleidomastoids were widely identified, and are key intrinsic factors for the required postures of optimal breastfeeding, this study supports existing literature regarding the role of MSK dysfunction in difficulties with breastfeeding. Given the importance of breastfeeding for short- and long-term health of both mother and infant, further high quality studies are required into the impact of MSK dysfunctions on suboptimal breastfeeding.

Ethical approval was granted from the AECC Research Ethics Subcommittee.

### P-03 Maternal report of breastfeeding outcomes following a single intervention in a combined chiropractic and midwifery breastfeeding clinic

#### Amy Miller^1^, Joyce Miller^2^, Alison Taylor^1^, Sue Way^1^

##### ^1^Bournemouth University, Poole, UK; ^2^Anglo-European College of Chiropractic (AECC), Bournemouth, UK

###### **Correspondence:** Amy Miller (amy.miller.chiro@gmail.com)

**Objectives**

Ability to breastfeed is one of the most crucial public health issues facing infants worldwide, and is particularly relevant given recent reports of the UK having the lowest sustained breastfeeding rate in the world. Effective means of supporting and improving breastfeeding should be a high healthcare priority. In this context, a combined chiropractic and midwifery clinic was developed to support mother-infant dyads struggling with suboptimal breastfeeding; however effectiveness of this approach has yet to be assessed. This study was designed to begin to address this question.

**Methods and materials**

Questionnaires were completed anonymously by mothers at presentation to the Interdisciplinary Breastfeeding Clinic. Data regarding the method of feeding, feeding experience of both mother and infant and common feeding difficulties were included. Postal follow up questionnaires were sent six to eight weeks after a single appointment at the clinic. Data were analysed using Microsoft Excel. The study took place between April 2015 and August 2016.

**Results**

301 mothers completed the intake questionnaire; 123 (41%) returned the postal follow up. The mean age was 5 weeks at intake and 15 weeks at follow up. At intake, 98.7% of infants were fed some breastmilk and 68.0% were totally breastfed. At follow up, 90.4% had some breastmilk and 64.2% were totally breastfed. Maternal experience of feeding was reported as a happy time by 24.3% of mothers at intake, increasing to 74.6% at follow up.

**Conclusion**

The results must be viewed in the current context of the dire breastfeeding rates in the UK, where only 23% of infants are exclusively breastfed at six weeks, with this figure dropping to 1% at six months. Within this study of dyads attending the IBC, breastfeeding was sustained at a far higher rate, providing support for the role of combined chiropractic and midwifery care for the problems of breastfeeding. Improving maternal experience of feeding is also an important factor in sustained breastfeeding. Whilst the design of this study does not allow these outcomes to be directly attributed to the intervention provided in the IBC, and it should be noted that mothers in this population were generally highly motivated, these results were positive and encourage further investigation. Additional study comparing mother-infant dyads attending the IBC with routine care would be beneficial to better understand the effect of this clinic.

Ethical approval was granted from the AECC Research Ethics Subcommittee.

### P-04 Outcome of low back pain patients referred from an Orthopedic Spine Unit to Chiropractic Treatment

#### Brigitte Wirth, Petra Schweinhardt, Kim Humphreys

##### Interdisciplinary Spinal Research, Department of Chiropractic Medicine, University Hospital Balgrist, Zurich, Switzerland

###### **Correspondence:** Brigitte Wirth (brigitte.wirth@balgrist.ch)

**Study Objectives**

In the setting of an orthopedic university hospital, the outcome of patients with low back pain (LBP) referred from the orthopedic spine unit to the division of Chiropractic Medicine was studied.

**Methods and Materials**

Between June 2014 and October 2016, 97 patients (54 male, mean age=45.6±16.5 years) from the spine unit were treated in the chiropractic policlinic. At baseline and after 1 week, 1, 3, 6 and 12 months, numeric rating scale (NRS) data for pain intensity and Bournemouth Questionnaire (BQ) data (bio-psycho-social outcome measure; maximum score=70 points) were collected. Additionally, the Patient’s Global Impression of Change (PGIC) scale was used at all time points apart from baseline. To depict the outcome, repeated measures ANOVA (post hoc Bonferroni correction) was used to describe the course of pain intensity and bio-psycho-social impairment. Furthermore, the proportion of patients reporting clinically relevant improvement in the PGIC (‘much better’ and ‘better’) was calculated for the patient group as a whole as well as for the acute/subacute (pain duration <3 months before treatment) and for the chronic subgroup.

**Results**

At baseline, mean NRS was 5.23 (±SD 2.54) and mean BQ was 38.79 (±SD 16.17) points. Pain intensity significantly decreased [F(3.96, 150.35)=11.16, p <0.001] to 3.36 (±SD 2.91) after 12 months (N=53). Similarly, bio-psycho-social impairment significantly diminished [F(3.41, 129.70)=17.64, p <0.001] to 24.47 (±SD 19.44) points (N=54). In the whole patient group, the proportion of patients reporting clinically relevant improvement increased from 27% (22/81 patients) after 1 week to 57% (44/77) after 3 months where it stabilized (56% after 6 months and 54% after 12 months). In the acute/subacute subgroup, clinically relevant improvement steadily increased from 39% (9/23) after 1 week to 79% (11/14) after 12 months. In the chronic subgroup, 22% (13/58) of the patients reported clinically relevant improvement after 1 week. This proportion increased to maximally 54% (31/57) after 3 months and then slightly decreased to 45% (17/38) after 1 year.

**Conclusions**

Despite the limitation of the lack of a control group, this study showed that chiropractic treatment was a valuable conservative treatment alternative to reduce pain and bio-psycho-social impairment in these mostly chronic patients who were referred from the surgeons in the orthopedic spine unit to chiropractic medicine. These findings underline the importance of collaboration between these two disciplines.

The ethics number is: EK-16/2009

Department of Chiropractic Medicine

University Hospital Balgrist

Forchstr. 340

8008 Zurich

Switzerland

### P-05 The immediate effects of Spinal Manipulative Therapy (SMT), in terms of performance tests assessment, namely in free squat and countermovement jump high (CMJ), on asymptomatic athletes. (Work In Progress)

#### Bruno A. P Alvarenga^1^, Marcelo B. Botelho^2^, Jerusa P. R. Lara^3^, António P. Veloso^4^

##### ^1^Lisbon University, FMH, Human Kinetic Faculty, 1499-002, Lisbon, Portugal; ^2^University of Bahia, Medicine Faculty, Salvador, 40026-010, Brazil; ^3^Federal University of Paraná, Curitiba, 80210-132, Brazil; ^4^Lisbon University, FMH, Human Kinetics Faculty, Biomechanics and Functional Morphology Laboratory, 1499-002, Lisbon, Portugal

###### **Correspondence:** Bruno A. P Alvarenga (brunofisioquiro@hotmail.com)

Currently, the sports practice, athletes have been suffering repeated with musculoskeletal problems, generating decrease in biomechanics parameters, affecting physical performance. This reality generates demand for technology and therapeutic options in favor of physical performance. [1] However very little is known about the biomechanics effect of (SMT) in terms of performance tests, on asymptomatic athletes. In our study the aims was quantitatively evaluate the immediate effects of (SMT), on performance tests. The technology used were through Biomechanics resources and therapeutic used, were through Spinal Manipulative Therapy (SMT). [1] The strategy used to measure physical performance was based on tests assessment with (SQT) Free Squat and Vertical Jump Countermovement (CMJ) High acting such a barometer of neuromusculoskeletal system. The therapeutic propose of SMT is, correcting vertebral dysfunctions through the application of high-velocity, controlled low-amplitude movement reducing internal mechanical stresses and restoring the restricted movements found along the spine, alter sensory and neurological signals improving physiological function.[2-3] To capture the kinematic and kinetic variables were used the motion caption system and the Qualisys Software, that collected through 15 sampling cameras at 179 frames per second, and 2 force platforms (left and right). The markers consisted of 49 reflective markers and 6 clusters, placed on participant body.[4] The sample size was 19 participants, randomly divided into groups, Group 1 (Lumbar SMT) and Group 2 (SHAM pre-positioning SMT). The preliminar intra-individual participant’s results, from ground reaction forces (GRF), showed considerable improvement on phase (Post), in strength symmetry of the lower limbs in Squat but still calculating the outcome results for propulsion phase of jump and Vertical Jump High (CMJ). The intra-individual and inter-individual results and conclusion, will be presented soon, after statistics analysis treatment.

**References**

[1] Theberge N. The integration of Chiropractors into healthcare teams: a case study from sport medicine. Sociology of Health & illness 2007, 30;1:19-34.

[2] DeVocht JW, Pickar JG, Wilder DG. Spinal manipulation alters electromyographic activity of paraspinal muscles: a descriptive study. J Manipulative PhysiolTher 2005, 28;7:465-471.

[3] Gal, J.M., Herzog, W., Kawchuk, G.N., Conway, P.J. and Zhang, Y.1994. Biomechanical studies of spinal manipulative therapy (SMT): quantifying the movements of vertebral bodies during SMT. Journal of the Canadian Chiropractic Association; 38: 11-24.

[4] Seay, J., Selbie, W.S., Hamill, J., 2008. In vivo lumbo- sacral forces and moments during constant speed running at different stride lengths. J. Sports Sci. 26, 1519–1529.

*FMH: registration number: #0577086

Ethics Committee from (FMH- Faculty of Human Kinetics), Lisbon, Portugal.

### P-06 Potential risk factors for persistent pelvic girdle pain 12 years postpartum

#### Cecilia Bergström^1^, Margareta Persson^2^, Ingrid Mogren^1^

##### ^1^Department of Clinical Sciences, Obstetrics and Gynecology, Umeå University, Umeå, Sweden; ^2^Department of Nursing, Umeå University, Umeå, Sweden

###### **Correspondence:** Cecilia Bergström (cecilia.bergstrom@umu.se)

**Objective**

Pelvic girdle pain (PGP) developed during pregnancy can persist long after delivery in some women. Nevertheless, for some women symptoms becomes more chronic in nature. Therefore, we aimed to identify potential risk factors for persistent pelvic girdle pain (PPGP) 12 years postpartum.

**Methods**

This is a long-term follow-up study based on a previous cohort of women reporting PGP during their pregnancy in 2002. Follow-up data was collected in 2014 and early 2015 through a new questionnaire that was sent out to a total of N=624 women. A modified Poisson regression as well as a modified Poisson autoregressive repeated measure analysis was performed to evaluate the relative risk (RR) of reporting PPGP 12 years postpartum.

**Results**

In total, 295 women (47.3%) responded to the questionnaire where n=36 (12.3%) reported ‘continuous’ pain, n=83 (28.3%) reported ‘recurrent’ pain, and n=174 (59.0%) reported ‘pain on a few occasions’/‘no’ pain. The RR of reporting pain 12 years postpartum based on the reported pain status at 6 and 14 months postpartum revealed a statistically significant increase in the RR of reporting pain 12 years postpartum if reporting ‘pain’ or ‘recurrent pain’ at 6 months after delivery (RR 1.86, p<0.0001 and 1.87, p<0.0001 respectively). Additionally, reporting ‘recurrent pain’ or ‘continuous pain’ 6 months postpartum demonstrated an increased RR of 1.79 (95% CI 1.21 – 2.63) and 4.24 (95% CI 1.30 – 13.89) of reporting ‘recurrent’ and ‘continuous’ pain 12 years postpartum respectively. However, as time progresses there was a statistically decrease in the long-term relative risk (RR) of PPGP between those who reported pain to different degrees and the ‘no pain’ group at all three time measures (6 months RR 0.46 (95% CI 0.41-0.51), 14 months RR 0.76 (95% CI 0.70-0.82), and 12 years RR 0.41 (95% CI 0.35-0.47) postpartum).

**Conclusion**

This unique longitudinal prospective study, following the natural course of PPGP, demonstrated that as time progresses there was a decrease in the long-term risk of PPGP up to 12 years postpartum. However, increased duration as well as persistency of PGP appears to be a major contributor of PPGP 12 years postpartum for a subgroup of women. Clinically, it would be valuable to identify women at risk of PPGP, especially given the adverse effects of PPGP on women’s health and the absence of effective treatment strategies during and after pregnancy.

Ethics Committee at the Umeå University (Dnr 2014-4-32M supplement to Dnr 2012-404-31M).

### P-07 Comparison of chiropractic treatment outcomes in neck pain patients depending on the sex of the chiropractor

#### B. Janine Thöni, Cynthia Peterson, B. Kim Humphreys

##### Department of Chiropractic Medicine, Faculty of Medicine, Orthopaedic University Hospital Balgrist, University of Zürich, Zürich, Switzerland

###### **Correspondence:** Cynthia Peterson (xraydcpeterson@yahoo.ca)

**Study Objective**

The high percentage of female chiropractic students in Switzerland suggests a future sex shift in the chiropractic profession in Switzerland. Thus it is necessary to identify if male and female chiropractors achieve the same treatment outcomes in neck pain patients. Therefore, the purpose of this study was to compare baseline factors and treatment outcomes in neck pain patients treated by male vs. female chiropractors.

**Methods and Materials**

This is a prospective outcome study including 849 patients with neck pain of any duration undergoing chiropractic treatment. Prior to the first treatment, baseline demographic data, the Bournemouth Questionnaire (BQ) and the numerical pain rating scale (NRS) for neck and arm pain were completed. At the follow-up time points of 1 week, 1, 3, 6 and 12 months, the Patient’s Global Impression of Change (PGIC) scale to categorize ‘improvement’ (primary outcome) and the BQ and the NRS for neck pain were completed. The PGIC includes 7 possible responses ranging from ‘much better’ to ‘much worse’. Only responses ‘much better’ and ‘better’ were considered ‘improved’. All other responses were considered ‘not improved’. Slightly worse, worse, and much worse were considered ‘worsening’ (secondary outcome). The Chi-square test compared the proportion of patients reporting ‘improvement’ or worsening between male and female chiropractors. The unpaired t-test compared the BQ and the NRS change scores between patients of male and female chiropractors at all time points.

**Results**

Patients of female chiropractors were more likely to report ‘improvement’ at 1 month (76.7% vs. 69.2%, p = 0.035) compared to male chiropractors and significantly more pain reduction at 3 months (NRS change score = 3.56 for female DC patients and 3.10 for male DC patients, p = 0.04). Patients of male chiropractors were significantly more likely to report ‘worsening’ at the 3 month time point (4.8% vs. 1.2%, p = 0.012). Patients of male chiropractors were older (43.15 years, vs. 39.71 years) (p = 0.0001), were less likely to have radiculopathy (11.2% of patients of male DCs compared to 17.3% for female DC patients, p = 0.014) and were less likely to take pain medication (27% of male DC patients vs. 35% of female DC patients, p = 0.046).

**Conclusions**

Improvement at 1 month and pain reduction at 3 months showed significantly better outcomes in patients of female chiropractors. Patients who were worse at 3 months were more likely to be treated by male chiropractors.

The ethics number is: EK-19/2009

KEK (Canton of Zurich ethics committee)

Balgrist Hospital ethics committee

### P-08 Cultural influence on treatment outcomes after chiropractic care in Swiss-German and Swiss-French patients: a prospective outcome study

#### David Guillén, Cynthia Peterson, B. Kim Humphreys

##### Department of Chiropractic Medicine, Faculty of Medicine, Orthopaedic University Hospital Balgrist, University of Zürich, Zürich, Switzerland

###### **Correspondence:** Cynthia Peterson (xraydcpeterson@yahoo.ca)

**Study Objective**

Culture is one of several aspects of the biopsychosocial model of low back pain (LBP). However, the analysis of cultural influences on pain is complex due to confounding factors (e.g. social- and educational status). Switzerland, with its well established social security system and homogenous educational system, meets optimal conditions for the research of language based cultural influence on LBP. The objective of this study was to compare outcome data and research the influence of ethno-cultural differences on low back pain treatment outcomes after chiropractic care in Switzerland between German and French speaking Swiss citizens.

**Methods and Materials**

This is a prospective outcome study including 1068 LBP patients. Baseline numerical rating scale for pain (NRS), demographic and Oswestry disability index (ODI) data were collected from low back pain patients presenting to 51 Swiss-German and 12 Swiss-French chiropractors. Treatment outcome data included the proportion of patients reporting clinically relevant ‘improvement’ on the Patient’s Global Impression of Change (PGIC) scale, and the NRS change scores collected at 1 week, 1, 3, 6 months and 1 year. ODI change scores were collected until 3 months. The proportion of patients ‘improved’ between the two groups was compared using the Chi2 test. NRS and ODI change scores were compared using the unpaired t-test.

**Results**

All baseline parameters, except for the age on the 853 German speaking Swiss and 215 French speaking Swiss patients revealed no significant differences. The PGIC, NRS and the ODI showed no significant differences between both patient groups up to 6 months. However, between the 6 month and 1 year time point the proportion of patients reporting clinically relevant ‘improvement’ continued to increase to 83.5% for the German speaking Swiss whereas it reduced to 73.1% for the French speaking Swiss (p = 0.01). The NRS change scores were also higher for the German speaking Swiss at 1 year compared to the Swiss-French citizens (p = 0.01).

**Conclusion**

Treatment outcome data after chiropractic care is comparable in the German and French parts of Switzerland until the 1 year time point when the French speaking Swiss patients are more likely to relapse. This may be due to cultural differences.

The ethics number is: EK-16/2009

KEK (Canton of Zurich ethics committee)

Balgrist Hospital ethics committee

### P-09 Chiropractic student perceptions of learning in inter-professional education: a qualitative study

#### Daniel Heritage, Joyce Miller

##### Anglo-European College of Chiropractic (AECC), Bournemouth, UK

###### **Correspondence:** Daniel Heritage (dheritage@aecc.ac.uk)

**Background**

There is growing interest in the development of inter-professional education (IPE), with the potential goal of achieving more effective healthcare delivery. It has been demonstrated that facilitators, students and patients can all benefit from IPE. However, IPE has been under-investigated in chiropractic education in Europe. Therefore, in a chiropractic teaching clinic (AECC), Bournemouth University midwives along with chiropractors developed an interdisciplinary new-born feeding clinic. Aims were to improve the feeding experience for mothers and infants with sub-optimal breast-feeding, to promote understanding between the two professions and encourage future relations between graduates.

**Methods**

Students from both courses spent one term each in the joint breastfeeding clinic. Students from both institutions were asked at the end of the academic year to attend a group semi-structured focus-group style interview to express their thoughts on the IPE experience. The focus group was conducted by an experienced researcher from Bournemouth University not previously known to the students. The sessions were recorded and transcribed. The group discussions were then analysed and themes from the discussions noted using word repetition analysis. This study focused on the views of the chiropractic students as the midwifery results were analysed by their own institution.

**Results**

All eligible students attended the session. Themes identified were educational value, understanding of another discipline, improved skills and better insight into the patient experience. Students expressed that there was excellent educational value from being a part of the joint feeding clinic. Chiropractic students felt they had greater understanding of the role of a midwife and how midwives approach sub-optimal breastfeeding, also a deeper understanding of their own work through questioning by the midwives. Students felt they obtained knowledge and skills (such as advanced history taking techniques) from the midwives that they could implement into their practice. Students also commented that they had gained a greater insight into the emotional strains mothers are under whilst struggling with breastfeeding. They expressed that parents felt very reassured when two professions agreed on the best course of action. Students believed the clinic helped develop their understanding of another profession and better understanding of the patient’s needs.

**Conclusion**

In an educational setting, the IPE worked well in practice. Chiropractic students felt that the experience was of significant benefit, particularly to develop their understanding of both the patients and another profession. Over-all they reported that the IPE greatly enhanced their education.

Bournemouth University Ethics Committee

Bournemouth University Ethics Committee provided ethics approval but do not provide a reference number. We did not necessarily require ethics approval as this was a group of students in a semi-structured interview who were fully aware of the point of the interview. However we applied for ethics approval out of good practice.

### P-10 A study to investigate final year chiropractic attitudes towards research based skills and competencies related to clinical practice

#### David Byfield, Annie Newsam

##### Welsh Institute of Chiropractic, University of South Wales, Pontypridd, UK

###### **Correspondence:** David Byfield (David.byfield@southwales.ac.uk)

**Study Objectives**

As the chiropractic profession moves towards its role as spine care experts, one of the important skills is the ability to implement guidelines and translate best evidence into clinical practice to improve patient care. In UK educational institutions delivering chiropractic awards, the research project has been viewed as the primary vehicle for students to demonstrate that they can apply the knowledge and skills of research and evaluation. The question arises, is the research project the most suitable instrument for students to fulfil these learning outcomes and provide the best method of inculcating the analytical skills to translate evidence into patient management. It is the view of the author that there are other educational approaches to introduce and assess these skills that are equally fit for purpose. It is becoming increasingly clear that chiropractors are essentially consumers and not necessarily generators of research. Consumers of research are defined by their ability to evaluate evidence and apply to clinical practice and thereby the care of patients, which is considered an important element of clinical competence.

The focus of undergraduate chiropractic education research should be fit for purpose for the clinical environment and that the emphasis on data collection should be shifted to postgraduate where time, funds and motivation are more appropriate. Models excluding primary data collection should focus on, clinical question generation, literature search skills, literature appraisal and critical and reflective cognitive skills and application of clinical research in practice to improve patient care.

In response to this alternative view of research and translating evidence into patient care, academic staff at the WIOC introduced a new research based module in 2016. This module included both a critical appraisal of an agreed peer reviewed scientific paper with clear operational criteria including a defense and written narrative and a clinic topic managed in the same rigorous fashion that would satisfy achieving these skills and complying with the GCC Educational Standards.

This poster will report the feedback gathered from final year students in terms of their confidence when critically appraising and applying the data into clinical practice and patient care. The poster will also include some qualitative data collected during the exercise as part of the overall student experience. This data will be used to inform future development of the module and this innovative method of applying and translating research data to clinical practice.

Ethical approval was gained via Faculty of Life Sciences and Education Ethics Committee.

### P-11 A study to investigate different approaches to learning in chiropractic students at the Welsh Institute of Chiropractic over two academic terms

#### David Byfield (David.byfield@southwales.ac.uk)

##### Welsh Institute of Chiropractic, University of South Wales, Pontypridd, UK

**Study Objectives**

A ‘learning style’ is described as the characteristics of a learner that influences the way in which that person learns and absorb new information and skills. Understanding the preferred learning style may help the learner to process, incorporate and accumulate new information and skills over the course of a professional degree programme. Each individual has their own diverse and distinctive characteristics that lead them to develop different learning styles. Knowing and understanding different learning styles can be helpful for students to improve their learning techniques and assist curriculum design and delivery. Therefore, the aim of this study was to continue to investigate how chiropractic students approach their learning and compare the responses across two academic years.

**Methods**

Students enrolled on years 2, 3 and 4 of the Master of Chiropractic programme were recruited for this study. They were requested to complete the Revised 20 question Study Process Questionnaire (R-SPQ-2F) to permit comparison with data collected in the previous academic year. Data from academic years 1, 2 & 3 from 2016 will be compared with academic years 2, 3 & 4 enrolled in 2017. The results will be tabulated as per the recommended scoring system to determine whether their learning preference was either, i) a Deep Learning Approach (Sum of all Deep Motive scores + all Deep Strategy scores) or ii) a Surface Learning Approach (Sum of all Surface Motive Scores + all Surface Strategy scores). Students were instructed to respond to the questionnaire based upon an overall view of their approach to their learning across the entire programme of study in their particular academic year and not individual modules.

**Results & Conclusions**

This poster will report the results of the learning style data collected from students enrolled on years 2, 3 and 4 of the Master of Chiropractic programme in 2017. The results will also include a comparison of overall learning style between cohorts sampled in 2016 to determine any changes in learning styles as students progress through the programme. Future studies will focus on specific modules within the programme to determine how students approach and learn the required knowledge and skills. This information could be quite valuable for the academic team during curriculum review to amend content, delivery mode and assessment strategy in order to achieve specific learning outcomes, attain clinical competencies and encourage a greater proportion of deep learning approach to chiropractic education.

Ethical approval was gained via Faculty of Life Sciences and Education Ethics Committee.

### P-12 Assessment of Symmetrigraph and Global Postural System Results for the Posture Analysis of the Healthy Individuals

#### Mehmet Toprak^1^, Hasan Kerem Alptekim^2^, Doruk Turhan^3^

##### ^1^Mehmet Toprak, Research Assistant, Bahcesehir University, Istanbul, Turkey; ^2^Hasan Kerem Alptekin, Assistant Professor, Bahcesehir University, Istanbul, Turkey; ^3^Doruk Turhan, Istanbul Kemerburgaz University, Istanbul, Turkey

###### **Correspondence:** Doruk Turhan (dorukturhan@gmail.com)

**Study Objectives**

Posture disorder is commonly seen in society. There are some differences among the reasons of them, these are ergonomi deficiency of office work environment, habits, cultural and sexual differences. The primer target of our work is to determine the similarities or differences of the methods by analysing the results of two of posture analysis methods used for the healthy individuals.

**Material and Methods**

In this study, the posture analysis has been made with Global Postural System and Symmetrigraph for 100 healthy individuals, 18-23 year-old, between the dates of March 2015- April 2015. Posterior and lateral posture analysis has been made for the individuals standing in front of the Symmetrigraph and Bragg posture table has been used for this analysis. Assessment of the posture has been made over triple scale. With the Global Postural System thoracic kyphosis and lumbar lordosis angles and measurements of sagittal plane head alignment have been calculated.

**Results**

Statistical analysis shows; in between symmetrigraph results of thoracic kyphosis and ages of the participants, there are not any meaningful differences. (p>0.05). As a result of the statistical analysis, the lumbar lordosis symmetrigraph results, there are meaningful changes with the aging of the individuals (p<0.05). Moreover, there are not any meaningful changes with the aging of the individuals on the head position in the sagittal plan in symmetrigraph method. (p>0.05). Only position of head in sagittal plan, results of both methods are compatible with each other. On the individuals 20 years and older, results are higher on symmetrigraph than global postural system for all perimeters.

**Conclusion**

In our study we have determined that the angle for thoracic kyphosis for the male individuals are lower than female individuals, female individuals have lumbar lordosis angle lower then male individuals while head position in sagittal plan has lower angle on the male individuals. When the results obtained from the studies are taken into consideration, it can be said that the results obtained from both methods do not show parallels in general. Consequently, we think that both methods can be used for the posture analysis, but the number and quality of the detailed studies related to this subject should be increased.

### P-13 Parent reported outcomes of infant care in a chiropractic teaching clinic versus private practices: a cohort study

#### Hazel Mellars, Joyce Miller

##### Anglo-European College of Chiropractic (AECC), Bournemouth, UK

###### **Correspondence:** Hazel Mellars (MellarsH@aecc.ac.uk)

**Study objectives**

Chiropractic care is an increasingly popular parental choice as a therapeutic intervention for infant conditions. Although patient-reported outcomes are a valuable tool in all types of healthcare to understand effects of treatment, usage for infant care has been minimal. Additionally there are virtually no observations on any differences between feasibility of use in teaching clinics versus private chiropractic clinics. This study implemented a parent reported outcome instrument for infant care in multiple chiropractic clinics to gather much needed data on the infant population along with any differences between types of clinics.

**Methods and materials**

A validated instrument (United Kingdom Infant Questionnaire) was implemented in the AECC teaching clinic and 15 private chiropractic clinics, using paper forms. New infant patients aged up to 12 months were included. Mothers completed the 12 item questionnaire which covered a range of common infant complaints to measure baseline characteristics prior to receiving chiropractic care. A 13 item follow-up questionnaire was completed at discharge, which included Parent Global Impression of Change (PGIC). SPSS. V. 21® was used in data analysis.

**Results**

A total of 521 infants from the AECC were included, and 51 from private clinics. Of these, 273 (52%) completed follow up forms at the AECC and 35 (69%) at private clinics. The most common complaint for patients at the AECC were feeding problems (40%) compared to private clinics with crying (47%). The average age at presentation to the AECC was 7 weeks compared with private clinics which was 15 weeks. The AECC had a mean satisfaction score of 9.6/10 (10 being completely satisfied) and the private clinics scored 8.5/10. Using only the top two ratings on the Parent Global Impression of Change (PGIC), at AECC 88% of infants had definite improvement compared to private practice with 38%. There were no adverse events reported, but at the AECC, 5% of parents reported mild side effects compared to 27% in private practice.

**Conclusion**

Parents reported good improvement along with high levels of satisfaction with care in all clinics. However, there was insufficient data from private clinics to make any representative conclusions about true similarities or differences between the teaching clinic and private chiropractic clinics. It was felt that the paper forms were too cumbersome for private clinics; further efforts should be made to encourage participation in private practice, such as electronic questionnaires. With wider usage, the UKIQ could be a practical tool to answer the need for outcome measures in paediatric practice.

Ethics:

The study was approved by the AECC Research Ethics Sub-Committee. All data were completely confidential. There was no way to identify any specific patient from any of the data collected in the forms. Only the members of the research team were able to access the questionnaires as they were stored in a locker in a card-access room, which was only accessible for authorized people, all in line with the Data Protection Act (1998).

### P-14 Do analogical models in teaching increase student learning?

#### Jacqueline Rix (jrix@aecc.ac.uk)

##### Anglo-European College of Chiropractic (AECC), Bournemouth, UK

**Introduction**

Lecturers frequently use analogical models to make abstract concepts more understandable to the students. These models are more than communication tools, they provide the student with a 3D tangible object for the means of exploring and describing anatomical and biomechanical subjects. However, lecturers cannot predict how a student will interact with a model. While it is recognised that the use of models enhance engagement, whether they are valuable epistemological tools remains to be seen. The aim of this block randomised control study was to look at whether analogical models provide some benefit to student learning.

**Method**

All Anglo-European College of Chiropractic students enrolled onto year one in 2014 and 2015 were eligible to take part. The study was carried out within the first two weeks of year one teaching, thus students from 2014 and 2015 had very similar teaching experiences within chiropractic education.Both groups received 3 one hour lectures on the topic of basic anatomy and biomechanics of the spine. The control group: 2014 student group received a PowerPoint presentation of the lectures only. The intervention group: 2015 student group received the identical PowerPoint presentation, with the addition of analogical models. The models included onions to demonstrate the layers of the intervertebral disc; jam doughnuts to demonstrate the disc and disc lesions; cardboard tubes to demonstrate the biomechanics of facet movement and liquorice allsorts to demonstrate creep and hysteresis. Both groups received an identical written assessment at the end of the third lecture.

**Results**

59 students from 2014 and 62 students from 2015 took part in the study. There were no significant differences in age or gender between the groups. The differences in the mean of the written assessment percentages between the control group and the intervention group were significant (p=0.00), with a mean difference and confidence interval (95%) of 22.625(17.376 – 27.874). The control group mean percentage was 37.93 (15.76) and is within a fail percentage bracket. The intervention group mean percentage was 60.56 (13.09) and is within a pass percentage bracket.

**Conclusion**

The students who received the PowerPoint lecture as well as the analogical models performed much better than the group that received the PowerPoint lecture alone. While student engagement clearly plays a part, using models as an epistemological tool cannot be ruled out. There is clearly a student learning advantage to tangible 3D models being used in lectures.

Ethics No. Analogical Models: E16/0911

The AECC Ethics Committee

### P-15 Time management and study skills as predictors of performance in a single cohort of undergraduate chiropractic students

#### Philip Dewhurst, Jacqueline Rix, Caroline Cooke, David Newell

##### Anglo-European College of Chiropractic (AECC), Bournemouth, UK

###### **Correspondence:** Jacqueline Rix (jrix@aecc.ac.uk)

**Objectives**

Predicting academic performance has largely been based on selection processes and previous academic performance. It is suggested that non-academic qualities such as time management and study methods are strongly linked to academic performance. This pragmatic study aims to examine student’s time management and study skills with respect to their summative assessment results. The intent is to look for early warning signs in students who are struggling in these areas, to enable early remediation to improve the students’ study success.

**Methods**

Students enrolled onto year one of the Chiropractic Masters (MChiro) at the Anglo-European College of Chiropractic were eligible to volunteer. Questionnaire data was collected at the beginning of the academic year; end of semester one and end of semester two. Semi-structured interviews were conducted at the end of semester one and end of semester two. Data collection was related to time management and study skills.

**Results**

At the beginning of the year students estimated how much time they would spend studying outside of timetabled teaching. This number was greater than the reported time spent in semester one, however reported time spent in semester two was significantly greater than both the estimate and the time spent in semester one. Each unit has an estimated number of hours a student should be studying, the time spent in semester one was less than this estimate, however time spent in semester two was significantly more than this estimate. Students who were unsuccessful in the summative assessments studied for significantly less time than their successful colleagues. 85.7% of students participated in extracurricular activities at the begging of the year. This reduced during semester one (74.4%) and again in semester two (62.8%). The students who stopped participating were significantly more successful in their assessments than those who continued to participate. 100% of students who stopped participating stated lack of time as their reason. There were no significant differences throughout the year regarding time spent with family, friends or social media. However, students suggested that their colleagues were their new family and friends. There were no significant changes to study methods throughout the year.

**Conclusion**

Before starting university, students had a preconceived idea of study time and methods. In semester one students struggled with time management, however following the semester one assessments students seemed to have a better understanding of the amount of studying required and successful students altered their extracurricular activities accordingly.

Ethics No. Predictors of Performance: E69/11/16

### P-16 The quality of life based on parent-child dyads using PROMIS

#### Joel Alcantara^1^, Jeanne Ohm^2^, Junjoe Alcantara^3^

##### ^1^The International Chiropractic Pediatric Association and Research Consultant, Sherman College of Chiropractic, Spartanburg, South Carolina, USA; ^2^The International Chiropractic Pediatric Association, Sherman College of Chiropractic, Spartanburg, South Carolina, USA; ^3^Private Practice of Chiropractic, Manila, Philippines

###### **Correspondence:** Joel Alcantara (dr_jalcantara@yahoo.com)

**Background**

It is acknowledged that agreement between self and proxy reports termed cross-informant variance is imperfect. The existence of cross-informant variance and the subjective nature of patient reported outcomes such as quality of life measures requires reliable and valid instruments. In terms of child self-reports, certain circumstances exist such as the child is too young, or too ill to complete a survey instrument, the parent proxy-report may be more applicable. Furthermore, parents’ perception of their child’s quality of life or state of health influences and determines healthcare utilization. Parent and child perspectives may be independently related to the kinds and types of healthcare used, risk factors, and quality of care and as such require further investigation.

**Objectives**

To investigate the quality of life of children and adolescents prior to a trial of chiropractic care using both paediatric and parent-proxy measures using PROMIS and determine their level of agreement.

**Methods**

To assess paediatric quality of life, children (age 8-17 years) under chiropractic care and their parent/guardian were asked to complete the PROMIS-25 and PROMIS proxy measures, respectively. Data analysis utilized the PROMIS manuals converting raw data to a T score metric (mean=50; SD=10). The greater the T score, the greater the measured QoL domain. Agreement of child and parent's proxy-report was assessed via intraclass correlation coefficient (ICC) via 2-way ANOVA.

**Results**

Two hundred and eighty nine parent/child pairings completed the questionnaires. The mean age of the children (164 females; 125 males) and parents (247 females; 42 males) were 12.52 years (SD=2.77) and 41.26 years (SD=6.88), respectively. The parents were highly educated with over 87% with some college or higher education. The majority (N=203) were presenting for wellness care and to relieve a symptom(s) followed by wellness care (N=47) and symptom care (N=39). Despite differences in mean T scores of parent versus child quality of life measures (i.e., mobility (50.82 vs. 49.91), anxiety (46.73 vs. 48.25), depression (45.18 vs. 55.63), fatigue (45.59 vs.43.27), peer relationship (52.15 vs. 51.2) and pain interference (47.47 vs. 46.85), agreement was fair with the following ICC values for mobility (0.46), anxiety (0.48), fatigue (0.43), peer and pain interference (0.48) while poor agreement for depression (0.12) and peer relationship (0.32).

**Conclusion**

There is fair agreement between parent/guardian and children’s rating of their QoL except for depression and peer relationship. Further research is supported given that parents decide the care of their child.

### P-17 The quality of life of chiropractic patients using the rand SF36

#### Joel Alcantara^1^, Jeanne Ohm^2^, Junjoe Alcantara^3^

##### ^1^The International Chiropractic Pediatric Association and Research Consultant, Sherman College of Chiropractic, Spartanburg, South Carolina, USA; ^2^The International Chiropractic Pediatric Association, Sherman College of Chiropractic, Spartanburg, South Carolina, USA; ^3^Private Practice of Chiropractic, Manila, Philippines

###### **Correspondence:** Joel Alcantara (dr_jalcantara@yahoo.com)

**Background**

It is imperative that evidence-based decision-making is based on high quality outcome measures. Well-defined and reliable PRO instruments can be used to support an intervention such as chiropractic. The RAND SF-36 is a set of generic, coherent, and easily administered quality-of-life (QoL) measure. It is widely utilized by managed care organizations and by Medicare for monitoring and assessment of US adult patients. However, to the best of our knowledge, the instrument has not been utilized in a large cohort of chiropractic patients.

**Objectives**

To investigate the quality of life of patients prior to a trial of chiropractic care using the RAND SF36.

**Methods**

Adult patients (≥18 years of age) attending care in a chiropractic practice-based research network completed a questionnaire inquiring on socio-demographic information, clinical correlates based on their history and physical examination and to complete the RAND SF36. The RAND SF-36 measures the domains of physical functioning, role limitation due to physical health, role limitation due to emotional problems, energy/fatigue, emotional wellbeing, social functioning, pain and general health.

**Results**

A total of 3247 patients (2242 females; 1005 males; mean age =42.48 years) comprised our study population. The subjects were highly educated with over 87% with some college or higher education. Interestingly, the majority of patients (61%) were currently experiencing a physical complaint with 19% concurrently receiving medical care while 39% did not currently experience a symptom. In terms of healthcare costs, 49% of patients paid for their care out of pocket, 12% solely by insurance and 39% a combination of both. The mean scores (range = 0-100) for our subjects in each of the quality of life domains along with a comparative measure to the Medical Outcomes Study (MOS) (N=2471) (i.e., DC patients vs. MOS Patients) are: physical functioning (81.91 vs. 70.61), role limitation due to physical health (70.59 vs. 52.97), role limitation due to emotional problems (72.05 vs. 65.78), energy/fatigue (56.84 vs. 52.15), emotional wellbeing (76.14 vs. 70.38), social functioning (80.56 vs. 78.77), pain (69.62 vs. 70.77) and general health (71.33 vs. 56.99). Overall, our chiropractic patients have a better quality of life than patients from the MOS survey. The Medical Outcomes Study: Measures of Quality of Life Core Survey (MOS) was a two-year study of medical patients with chronic conditions.

**Conclusion**

Patients in this study present for chiropractic care with an overall better quality of life than medical patients from the MOS study.

### P-18 Service evaluation of health seeking behaviour in spinal patients in the 12 months after discharge from a state funded chiropractic service

#### Jonathan Field^1,2^, Dave Newell^1^

##### ^1^Back2Health, Petersfield, UK; ^2^Anglo-European College of Chiropractic (AECC), Bournemouth, UK

###### **Correspondence:** Jonathan Field (jonathanfield@me.com)

Spinal pain is a common and costly problem for society. High recurrence rates are a cause of concern for those commissioning services with patients perceived as having either repeated cycles of care or going onto to have more interventional and expensive hospital treatment. One previous study has reported that care seeking for future episodes of spinal pain is higher amongst patients who had initially seen a chiropractor. In the UK there are examples of NHS state funded chiropractic services being available to patients via GP referral. If a goal of care is to better equip patients to manage flare-ups understanding how these services perform is important to inform future commissioning decisions and assist service development. The presented study sought to explore the health seeking behaviour in a population of spinal pain patients who had been discharged to the community (not needing further care) following a treatment with a Back2Health, a NHS funded chiropractic service. Discharges over a two month period were reviewed. Those reported as not needing further care were telephoned a year later and asked about recurrence and health seeking behaviour. Contact was made with 92 patients (46%), one not wishing to participate. 63% reporting a flare-up of symptoms they would rate as ‘troublesome’. Of these 29.6% had sought further care (GP 14.3%, Hospital services 8.2%, Chiropractor 4.1% & Physiotherapist 4.1%). As in previous studies the rate of reported flare-up was high, however many experiencing a return of symptoms felt able to self-manage. One third of the total population had sought further care in the 12 months after discharge, with half of this being from their GP and a quarter seen in hospital. Access to this service was via GP referral, and GPs were precluded from making a repeated referral to the same provider twice in a year. It is possible that this may have encouraged a higher proportion of hospital referrals in patients who otherwise may have been seen in the community. In a previous report these authors have described the positive health outcomes and experiences of a substantial proportion patients attending NHS chiropractic care. However the presented data suggests that a significant number of patients go on to have further care in the year following discharge. Further work is strongly indicated in this area. The low proportion of patients contacted is a limitation of this study. It is possible that other services may see different results.

This study was deemed not to require ethics approval by NIHR as it was a ‘service evaluation’ and not a research project

### P-19 An electronic parent reported infant outcome measure in chiropractic clinics: a feasibility study

#### Heather Hanson, Joyce Miller

##### Anglo-European College of Chiropractic (AECC), Bournemouth, UK

###### **Correspondence:** Joyce Miller (jmiller@aecc.ac.uk)

**Background**

The United Kingdom Infant Questionnaire (UKIQ) is a validated parent reported outcome measure based on common public health issues and presenting complaints of infancy. The UKIQ is proposed as a pragmatic tool for research purposes and an electronic version might be advantageous for large-scale data collection in chiropractic clinics.

**Study objectives**

The primary objective of this study was to test the feasibility of an electronic version of an infant outcomes instrument. To this end, feasibility benchmarks were set based on technical performance and acceptance of the tool by its users, chiropractic offices and mothers.

**Materials and methods**

In this prospective, multi-centre observational study, mothers of infants (0-12 months) were asked to complete the electronic UKIQ at initial presentation and at follow-up in chiropractic clinics in the United Kingdom. Technical issues encountered and rates of non-consent were tracked during the study period. Feasibility benchmarks included the following: recruitment of four participating clinics, a minimum of 70% maternal consent to participation, 80% completeness of data, 80% response from mothers that the questionnaire was “easy to use” and a follow-up rate of 50%. Participating chiropractors and receptionists were asked for feedback and ratings of their experience with the tool. Additionally, clinics who showed interest in participating but did not participate were asked to provide feedback in order to elucidate barriers to implementation.

**Results**

During the testing period, 102 intake and 46 follow-up forms were completed. All feasibility benchmarks were achieved, except for follow-up rate (46%) and lower than anticipated participation of recruited clinics (n=2). No technical difficulties prevented mothers from completing the form and collected data had a high rate of completeness with <0.03% missing data for standard questions and no undecipherable answers. No incidents of maternal non-consent occurred and 98.9% of mothers found the tool easy to use. Clinicians and receptionists rated their experience with the tool highly (mean 5/5 for clinician perception of clinical utility and likelihood of continued use and mean 4.5/5 for receptionist’s ease of administering and 5/5 for receptionist perception of willingness of mothers to complete and ease with which mothers completed the questionnaire).

**Conclusions**

This tool is technically capable of large-scale data collection and well-accepted by users. However, implementation of the instrument in private chiropractic clinics was low. Future research must investigate methods to improve uptake of key outcomes instruments in chiropractic practices.

Anglo European College Research Ethics Sub-Committee.

### P-20 Portable pad or pen and paper? Preference of mothers completing outcomes instrument: a cross-sectional survey

#### Hiew Mandy, Lo Derek, Mok Zicheng, Tee Yun Han, Miller Joyce

##### Anglo-European College of Chiropractic (AECC), Bournemouth, UK

###### **Correspondence:** Miller Joyce (jmiller@aecc.ac.uk)

**Background**

Outcome instruments are widely used as a quantitative measurement of a patient’s overall status by health care practitioners as well as an evaluator of the patient’s health during an episode of care. Traditionally, these instruments were paper-based and kept in the patient’s file for the doctor to reference over time. Due to advancing technology, paperless offices have adopted environmentally-friendly initiatives and efficiencies where electronic forms are preferred. However, it is unknown whether the consumer concurs. A Canadian study of 45 patients who were given options of questionnaires in either paper or electronic format showed preferences for the software version. Another type of preference study in Scandinavia compared the response rates of parents of children using four different modes of data collection (paper, web, web and paper, web with incentive). Their result showed no loss of data regardless of the collection method. No studies have been done to compare paper or electronic preference on the specific population of parents reporting outcomes for their infant.

The objective of this study was to investigate the preferred method of collection, paper or electronic, for mothers completing a validated infant outcomes instrument, the United Kingdom Infant Questionnaire (UKIQ) who presented their baby for treatment at a chiropractic teaching clinic on the south coast of England.

**Methods**

Mothers of children aged 0-12months were asked to complete either the intake or follow-up questionnaire in both paper and electronic formats. The mother was then given another form asking her preference for type of questionnaire. There was an opportunity for her to write comments on the forms if she wished. The responses were collected and collated by the research team, using Excel for descriptive statistics.

**Results**

In all, 46 mothers participated; 32 (70%) preferred the electronic questionnaire, 9 (19%) preferred the paper version and 5 (11%) felt neither one was better than the other. Comments included that the electronic form was easier, quicker and it was better to save the trees.

**Conclusions**

Although the number was small, this study showed that electronic-based questionnaires were preferred by mothers of infants as they find it easier and faster to complete. It is logical that mothers choose web-based forms as these are young women who are accustomed to this type of technology. With the progression of technology, electronic questionnaires could be considered a more efficient method for collecting important data, without loss due to patient or parent preferences.

AECC Projects Panel.

### P-21 Description of musculoskeletal extremity complaints in children and adolescents: a systematic review

#### Signe Fuglkjær^1^, Kristina Boe Dissing^1^, Lise Hestbæk^1,2^

##### ^1^Department of Sports Science and Clinical Biomechanics, Faculty of Health Sciences, University of Southern Denmark, Campusvej 55, DK-5230 Odense M, Denmark; ^2^Nordic Institute of Chiropractic and Clinical Biomechanics, Campusvej 55, DK-5230 Odense M, Denmark

###### **Correspondence:** Signe Fuglkjær (sfuglkjaer@health.sdu.dk)

**Background**

It is difficult to gain an overview of musculoskeletal extremity complaints in childhood although this is essential to develop evidence-based prevention and treatment strategies. The objectives of this systematic review were therefore to describe the occurrence of musculoskeletal extremity complaints in children and adolescents in both general and clinical populations in relation to age, anatomical site and mode of onset.

**Methods**

MEDLINE and EMBASE were electronically searched; risk of bias was assessed; and data extraction was individually performed by two authors.

**Results**

In total, 19 general population studies and three clinical population studies were included. Due to heterogeneity of the included studies an overall estimate of prevalence or incidence was not possible. Lower extremity complaints were more common than upper extremity complaints regardless of age and type of population, with the most frequent pain site changing from ankle/foot in the youngest to knee in the oldest. There were about twice as many non-traumatic as traumatic complaints in the lower extremities, whereas the opposite relationship was found for the upper extremities in the general population studies. There were relatively more lower extremity complaints in the general population studies than in the clinical population studies. The review showed no pattern of differences in reporting between studies of high and low risk of bias.

**Conclusions**

This review shows that musculoskeletal extremity complaints are more frequent in the lower extremities than in the upper extremities in childhood, and there are indications of a large amount of non-traumatic low intensity complaints in the population that do not reach threshold for consultation.

### P-22 Does it matter for outcome *how chronic* chronic pain is?

#### P. Schweinhardt^1,2^, B. Wirth^1,2^, G. Peterson^1,2^, B. K. Humphreys^1,2^

##### ^1^University Hospital Balgrist, Zürich, Switzerland; ^2^Zurich University, Zürich, Switzerland

###### **Correspondence:** P. Schweinhardt (Petra.Schweinhardt@balgrist.ch)

**Aims**

Patients with pain complaints longer than three months (or six, depending on definition employed) are classified as ‘chronic’. Outcome in chronic pain patients is less favourable compared to acute or sub-acute patients. However, it is currently unknown whether symptom duration matters for outcome once pain is chronified. Here, we investigated whether symptom duration influenced outcome in patients with chronic (>3 months) low back pain.

**Methods**

Data collection was performed between 2010 and 2014 among 66 chiropractors in Switzerland. Treatment was performed as per routine chiropractic practice. Patients’ pain levels were assessed at baseline, 1 week, 1 month, 3, 6, and 12 months using an 11-point numerical rating scale (NRS). Patient’s Global Impression of Change (PGIC) was collected at 1 week, 1 month, 3, 6, and 12 months. Duration of the present pain complaint served as independent variable. Its influence on three-month PGIC scores as well as on measures arguably less vulnerable to response bias (‘other doctors seen (for low back pain) since last assessment’ and ‘time since last chiropractic treatment’) was evaluated using univariate Analyses of Variance (ANOVA). Further, to reduce the likelihood that response bias drives PGIC scores (patients answering positively when asked on the phone), we excluded patients in whom PGIC and NRS change scores were inconsistent (i.e. increase on NRS from baseline to 3 months and PGIC indicating improvement or decrease on NRS and PGIC indicating worsening) in a second analysis.

**Results**

367 (/1101) of participating patients presented with pain durations > 3 months; for 237 of those the exact durations were available. Pain duration followed a right-skewed distribution with 34.6% > 3 months and ≤ 12 months, 25.7% > 12 months and ≤ 24 months, and 17.3% > 60 months (up to 30 years). According to PGIC scores, 68% reported clinically relevant improvement (‘very much improved’ or ‘much improved’) at three months. This number remained stable at 6 and 12 months (68% at all time points). Pain duration did not influence patients’ outcome whether assessed by PGIC, ‘other doctors seen’ or ‘time since last chiropractic treatment’ (p’s>0.2). In the patients with consistent PGIC and NRS change scores (n=171), there was neither an influence of pain duration on outcome measures (p’s>0.2).

**Conclusions**

In patients with low back pain longer than three months, the likelihood to improve with chiropractic treatment did not depend on symptom duration. Even in patients with very longstanding pain, improvement was considerable.

The Ethics number is: EK-16/2009

Kantonale Ethikkomission Zurich

### P-23 Common drive and self-sustained firing in cervical multifidus motoneurons in neck pain patients and healthy subjects

#### Tim L. Raven, Lise R. Lothe, Torsten Eken

##### Department of Anaesthesiology, Oslo University Hospital (OUH), University of Oslo, Oslo, Norway

###### **Correspondence:** Tim L. Raven (raventim@gmail.com)

**Background**

Neck pain (NP) causes deterioration of paraspinal muscle function, however the underlying neurophysiology of this phenomenon is poorly understood. The cervical multifidus (CM) muscle has been implicated in NP. We aimed to investigate the degree of coordination of motoneuron activity in CM in subjects with NP compared to healthy controls (HC), specifically (1) the correlation between motor unit (MU) discharges as a measure of the common drive to the motoneuron pool, and (2) signs of self-sustained firing and rotation of activity between MUs.

**Methods**

Fine-wire electromyography (EMG) electrodes were implanted bilaterally into CM in 10 NP subjects and 15 HC under CT guidance. Paired recordings of simultaneously discharging MUs were used to determine the degree of cross correlation of neuronal drive as measured by the common drive coefficient (CDC). Effects of a number of independent variables on CDC between concurrently active MUs were assessed using linear mixed models.

**Results**

Viable EMG data was available from 22 electrodes in 13 of the 15 HC, and from 17 electrodes in 9 of the 10 NP subjects providing 348 unilateral and 263 bilateral MU pairs. The final mixed model demonstrated lower CDC in bilateral vs. unilateral MU pairs (0.11; 95% CI 0.09 to 0.14; P <0.0001), and in NP vs. HC subjects (0.13; 95% CI 0.05 to 0.21; P = 0.003). CDC was also lower in MU pairs during sitting vs. standing (0.07; 95% CI 0.04 to 0.10; P <0.0001), and lower in voluntary vs. spontaneous activity (0.07; 95% CI 0.02 to 0.11; P = 0.004). R2 = 0.51 for the entire model.

We observed in both HC and NP subjects that MUs were recruited from inactivity to tonic discharge lasting for several minutes without changes in the discharge rate in other already active units. Several instances were documented where activity was rotated between MUs.

**Discussion and Conclusions**

Motoneurons to CM are under the influence of varying degrees of common drive depending on the postural task, with largest common drive occurring in unilateral MU pairs during spontaneous standing. Different strategies were in play when attempting to keep a fixed force output as opposed to when force was kept constant unconsciously, and these strategies were altered in the presence of NP. Long-lasting activity within narrow firing rate ranges was rotated between MUs, which we suggest was probably due to activation and inactivation of self-sustained discharge in individual motoneurons.

Ethical approval for the study was granted by the Regional Ethics Committee South-Eastern Norway, (ref. S-09077d, 2009/374).

### P-24 The central role of the sacroiliac joint in musculoskeletal dysfunctions

#### Rick Serola (rick@serola.net)

##### Founder and Co-CEO of Serola Biomechanics, Inc, Loves Park, Illinois, USA

**Background**

Historically, it was thought that muscles did not move the sacroiliac joint (SIJ); instead, movement was supposedly caused by axial forces during trunk movement. Although muscles have recently been shown to move the SIJ, this concept has attained only minor relevance in diagnostic protocols. The goal of this article is to help conceptualize the possible wide-ranging effects of SIJ dysfunction on musculoskeletal syndromes.

**Methods**

Multiple Pub Med literature searches were made for search terms such as “sacral nutation AND musc*,” “sacral nutation and kinematic chain,” “sacroiliac and musc*, pelvic floor musc*.” Abstracts were reviewed to determine which papers were pertinent for this review.

**Results**

The literature suggests that, because the SIJ is positioned at the center of movement, shock absorption, and load transfer, it functions as our structural core. As in any joint, the muscles that attach to the pelvis are regulated by the ligaments within the SIJ. Accordingly, the vast array of SIJ ligaments may have a direct correlation to the extensive number of muscles that move the joint, which suggests that the majority of our musculoskeletal system may be directly affected by the SIJ. Consequently, the muscles that attach to the pelvis may play a key role in integrating the musculoskeletal system through sacral nutation and counternutation patterns, which may influence the entire kinematic chain.

Axial force directed inferiorly through the spine or superiorly through the legs will induce SIJ nutation. When the force exceeds the ligaments’ ability to maintain integrity, they sprain. The literature suggests that, in order to avoid overstressing the sprained ligaments, protective ligamento-muscular reflexes are initiated, conceivably activating the counternutation muscles and inhibiting the nutation muscles on the side of injury.

Once injured, ligaments often tend to heal poorly and may subsequently create a long term compensatory adaptation process affecting the kinematic chain. An SIJ sprain may change coordination patterns in muscles which, by their proximal attachments to the pelvis and distal attachments to the spine and lower extremities, may alter posture and joint angles throughout the body.

**Conclusion**

An SIJ sprain may result in ligamento-muscular responses that may underlie many dysfunctions of the spine, pelvis, and extremities; the pelvis may torque, the spine may twist, and the extremities may rotate, and lead to asymmetrical movement patterns. More research is needed to study the prevalence of the SIJ nutation lesion in musculoskeletal injuries.

