# Correction to: P-12 Assessment of Symmetrigraph and Global Postural System Results for the Posture Analysis of the Healthy Individuals

**DOI:** 10.1186/s12998-018-0190-2

**Published:** 2018-04-27

**Authors:** Mehmet Toprak, Hasan Kerem Alptekin, Doruk Turhan

**Affiliations:** 1 Bahcesehir University, Istanbul, Turkey; 2 Bahcesehir University, Istanbul, Turkey; 3 Istanbul Kemerburgaz University, Istanbul, Turkey

## Correction

After publication of the below abstract in supplement [1] it was bought to our attention that in abstract P-12, author Kerem Alptekin’s surname name had been published incorrectly as Alptekim. The authors also wished to add the below information to the abstract:

Ethics Committee of Clinical Researches at Bahcesehir University

No: 22481095.00.00.00.00.00.00.00-604.01.02-231

Date: 30/04/2015

The full and corrected abstract has been included below:

**P-12 Assessment of Symmetrigraph and Global Postural System Results for the Posture Analysis of the Healthy Individuals**

Mehmet Toprak^1^, Hasan Kerem Alptekin^2^, Doruk Turhan^3^

^1^Mehmet Toprak, Research Assistant, Bahcesehir University, Istanbul, Turkey; ^2^Hasan Kerem Alptekin, Assistant Professor, Bahcesehir University, Istanbul, Turkey; ^3^Doruk Turhan, Istanbul Kemerburgaz University, Istanbul, Turkey

**Correspondence**: Doruk Turhan (dorukturhan@gmail.com)

### Study Objectives

Posture disorder is commonly seen in society. There are some differences among the reasons of them, these are ergonomi deficiency of office work environment, habits, cultural and sexual differences. The primer target of our work is to determine the similarities or differences of the methods by analysing the results of two of posture analysis methods used for the healthy individuals.

### Materials and Methods

In this study, the posture analysis has been made with Global Postural System and Symmetrigraph for 100 healthy individuals, 18-23 year-old, between the dates of March 2015- April 2015. Posterior and lateral posture analysis has been made for the individuals standing in front of the Symmetrigraph and Bragg posture table has been used for this analysis. Assessment of the posture has been made over triple scale. With the Global Postural System thoracic kyphosis and lumbar lordosis angles and measurements of sagittal plane head alignment have been calculated.

### Results

Statistical analysis shows; in between symmetrigraph results of thoracic kyphosis and ages of the participants, there are not any meaningful differences. (p>0.05). As a result of the statistical analysis, the lumbar lordosis symmetrigraph results, there are meaningful changes with the aging of the individuals (p<0.05). Moreover, there are not any meaningful changes with the aging of the individuals on the head position in the sagittal plan in symmetrigraph method. (p>0.05). Only position of head in sagittal plan, results of both methods are compatible with each other. On the individuals 20 years and older, results are higher on symmetrigraph than global postural system for all perimeters.

### Conclusion

In our study we have determined that the angle for thoracic kyphosis for the male individuals are lower than female individuals, female individuals have lumbar lordosis angle lower then male individuals while head position in sagittal plan has lower angle on the male individuals. When the results obtained from the studies are taken into consideration, it can be said that the results obtained from both methods do not show parallels in general. Consequently, we think that both methods can be used for the posture analysis, but the number and quality of the detailed studies related to this subject should be increased.

Ethics Committee of Clinical Researches at Bahcesehir University

No: 22481095.00.00.00.00.00.00.00-604.01.02-231

Date: 30/04/2015
